# Effect of Bean-Derived Soluble Dietary Fibers on Macrophage Function In Vitro

**DOI:** 10.3390/foods15091471

**Published:** 2026-04-23

**Authors:** Ana M. Magallanes López, Mark Williamson, Senay Simsek, Estelle Leclerc

**Affiliations:** 1 Department of Plant Sciences, North Dakota State University, Fargo, ND 58105, USA; 2 School of Medicine and Health Sciences, University of North Dakota, Grand Forks, ND 58202, USA; mark.williamson.2@und.edu; 3 Whistler Center for Carbohydrate Research, Department of Food Sciences, Purdue University, West Lafayette, IN 47907, USA; 4 Department of Pharmaceutical Sciences, North Dakota State University, Fargo, ND 58105, USA

**Keywords:** dietary fiber, dry bean, macrophage function

## Abstract

Studies have shown that dietary fibers have many health benefits. Soluble dietary fibers (SDF) extracted from wheat, corn, rice, or several herbaceous plants have been shown to have either pro- or anti-inflammatory effects depending on the mode of preparation of the fibers, the fibers’ structures and the biological or cellular context. However, much less is known regarding the immunomodulatory properties of dry bean-derived SDF. The goal of this study was to fill this gap in knowledge. Using RAW 264.7 macrophages, we show that dry bean-derived SDF stimulated the production of nitric oxide (NO), tumor necrosis factor (TNF) α, interleukin (IL)-1β and IL-6. We show that these changes were partly dependent on toll-like receptor TLR-4 signaling. More importantly, we observed that the levels of NO, TNF-α, IL-1β and IL-6 were significantly lower when the SDF were extracted from heat-processed bean flour. Overall, our results demonstrate that dry bean-derived SDF-rich fractions modulate macrophage activation in vitro, promoting a pro-inflammatory response that is partially mediated by TLR-4 signaling.

## 1. Introduction

Dietary fiber (DF) originates from cereals, legumes, fruits, nuts, seaweed or vegetables and is a collective term to describe carbohydrate polymers that are neither absorbed nor hydrolyzed in the small intestine and are nearly or completely fermented in the colon [1,2,3]. Dietary fiber is divided into two groups: insoluble dietary fiber (IDF), which includes lignin, cellulose and hemicellulose, and soluble dietary fiber (SDF), which includes pectin, gums, mucilages, and inulin-type fructans [4].

In recent years, dietary fibers from plants have been recognized for their many health benefits [5,6]. They improve digestion through their laxative effects by increasing fecal bulking, softening, frequency and regularity [7]. They also have beneficial effects on the cardiovascular system by reducing the risks of coronary heart disease [8,9], hypertension and stroke [10,11], diabetes and obesity [11,12,13].

The role of plant-derived dietary fiber on the function of the immune system has also been explored, and there is currently consensus that dietary fiber has immunomodulatory effects [14,15,16,17]. It was found that dietary fiber could have either pro- or anti-inflammatory effects, depending on the source of the fiber, the modes of preparation and structures and the biological or cellular context [17,18,19,20]. For example, rice bran-derived arabinoxylans showed pro-inflammatory effects by enhancing natural killer (NK) cell activity and dendritic cell maturation when injected in mice [21,22]. Similarly, polysaccharides derived from *Platycodon grandiflorum*, a flowering perennial plant, were shown to induce the maturation of dendritic cells and the concurrent increase in IL-2, tumor necrosis factor (TNF) α, interleukin (IL)-1β, IL-6, IL-10 and interferon (INF)-β [23]. Increases in the levels of pro-inflammatory cytokines were also observed in murine macrophages stimulated by polysaccharides isolated from *Tinospora cordifolia* [24]. In a different study, Li et al. also showed that wheat-derived arabinoxylan extracts could stimulate the nitric oxide (NO) production by U937 monocytes to levels similar to those produced after stimulation with lipopolysaccharides (LPSs) [20].

As mentioned above, many studies have investigated the immunomodulatory effects of SDF from wheat, corn, rice, and several herbaceous plants. However, the health benefits of dry bean-derived SDF have been relatively underexplored. The recent study of Wu et al. compared the biological functions of ten traditional legumes and showed that, in their experimental conditions, all SDF presented potential antioxidant and antiglycation effects, as well as stimulated the production of NO, TNF-α and IL-6 from RAW macrophages [25]. Our present study further demonstrates the activation of RAW macrophages by dry bean-derived SDF. We show that the treatment of RAW cells with SDF extracted from dry bean flour resulted in increased production of NO, TNF-α, IL-6, and IL-1β. Notably, using an inhibitor of TLR-4, we showed that the increased levels of NO and of the pro-inflammatory cytokines were in part dependent on the TLR-4 signaling pathway. In addition, we observed that heat processing the dry bean flour prior to extracting the SDF resulted in reduced levels of both NO and pro-inflammatory cytokines produced by the RAW macrophages.

## 2. Materials and Methods

### 2.1. Materials

The RAW 264.7 murine macrophage cell line was purchased from the American Type Culture Collection (ATCC) (Manassas, VA, USA). Dulbecco’s Modified Eagle’s Medium (DMEM) (ATCC^®^. 30-2002) and Fetal Bovine Serum (FBS) (ATCC^®^. 30-2020) were purchased from ATCC. Trypsin (0.25%) EDTA (1×) (VWR, 02-0154-0100) was purchased from VWR (Solon, OH, USA). LPS from *E. coli*, Serotype O111:B4 (1 mg/mL, ALX-581-012-L002) was obtained from Enzo Life Sciences (Farmingdale, NY, USA). Tak-242 (TLR 4 signaling inhibitor, A3850) was purchased from APExBIO (Houston, TX, USA). Genscript ToxinSensor™ Chromogenic limulus amebocyte lysate (LAL) Endotoxin Assay Kit (L00350) was purchased from Genscript (Piscataway, NJ, USA). The mouse TNF-α Quantikine ELISA kit (MTA00B), mouse IL-6 Quantikine ELISA kit (M6000B), and mouse IL-1β Quantikine ELISA kit (MLB00C) were purchased from R&D Systems, Inc. (Minneapolis, MN, USA). The Griess Reagent System (G2930) was obtained from Promega Co. (Madison, WI, USA). The PureLink RNA Mini Kit (12183018A) was obtained from Invitrogen (Waltham, MA, USA). The First Strand cDNA Synthesis (Quick Protocol) (M0253) components were purchased from New England BioLabs Inc. (Ipswich, MA, USA). The Brilliant II SYBR^®^ Green QRT-PCR Master Mix with Low ROX was obtained from Agilent Technologies (Santa Clara, CA, USA).

### 2.2. Production of SDF Fractions

Two varieties of dry beans, Monterrey (pinto bean) and Eclipse (black bean), were used in the study. Both varieties were grown in North Dakota (ND). Monterrey is a high-yielding variety resistant to common bean diseases, while Eclipse was the first black bean variety released by the ND Agricultural Experiment Station; both are agriculturally relevant and were chosen to represent compositional diversity between pinto and black bean types. Before further processing, the beans were first cleaned by removing foreign material and damaged seeds. The flour used for the SDF extraction came from non-dehulled beans to analyze the bean components of the whole seed.

The SDF fractions were prepared from the flours by the enzymatic-gravimetric procedure according to Feng et al. with slight modifications [26]. Briefly, the flour samples underwent stepwise enzymatic hydrolysis of α-amylase, protease, and amyloglucosidase. After filtration, the hydrolyzate filtrates were collected and concentrated to about one-tenth in a vacuum rotary evaporation system. The SDF fractions were obtained by ethanol precipitation followed by freeze-dry processing [27]. Six different batches of SDF were prepared from cooked Pinto/Monterrey bean flour, six from cooked Black/Eclipse bean flour, six from raw Pinto/Monterrey bean flour, and six from raw Black/Eclipse bean flour.

### 2.3. Determination of Endotoxin Level in SDF Samples

In this study, it was essential to ensure that the dietary fiber preparation was not contaminated by endotoxins. For this reason, the level of endotoxin in each 500 µg/mL SDF sample was determined, prior to performing the cell-based assays, using the ToxinSensor^TM^ Chromogenic LAL Endotoxin Assay according to the manufacturer’s instructions. The experiment was performed using technical duplicates.

### 2.4. Determination of Immunomodulatory Properties of SDF Samples

The RAW 264.7 murine macrophage cell line was chosen because it has been used extensively to evaluate the immunomodulatory effects of plant-derived extracts [18,24,28,29,30,31,32,33]. RAW 264.7 cells have high plasticity and can be polarized into either the pro-inflammatory M1 type or the anti-inflammatory M2 type, depending on the stimulants. M1 type macrophages typically produce NO, pro-inflammatory cytokines including TNF-α, IL-1β and IL-6 [34]. For all these reasons, this cell line was appropriate to study the immunomodulatory effects of dry bean-derived SDF. RAW 264.7 cells, referred to as RAW cells, were grown in DMEM supplemented with 10% FBS and 1% penicillin-streptomycin solution (hereafter referred to as complete media) at 37 °C under a humidified atmosphere of 95% air and 5% CO_2_. The cells were cultured in complete media until they reached 90% confluency. Trypan blue (Mediatech, Inc., Manassas, VA, USA) staining (0.4% (*w*/*v*) was performed to assess the viability of cells at times of cell seeding. Cells were counted using a hemocytometer. RAW cells were seeded at 3 × 10^5^ cells per well in the presence of 600 mL complete media, in 24-well plates. After attachment, the cells were treated with SDF (500 µg/mL). This concentration was chosen based on our previous studies using wheat-derived SDF and initial testing [18]. Untreated cells were used as negative controls. Cells treated with 1 µg/mL LPS were used as positive controls. To block TLR-4 signaling, when indicated, cells were treated with 10 µM Tak-242 prior to being treated with the SDF. Because Tak-242 was prepared in DMSO, an additional negative control (0.1% DMSO) was used.

### 2.5. Determination of TNF-α, IL-6 and IL-1β by ELISA

TNF-α, IL-6 and IL-1β levels were measured from cell culture supernatants using ELISA kits following the manufacturer’s instructions. The supernatants from SDF-stimulated cells were diluted 10 times prior to measuring the TNF-α and IL-6 levels by ELISA. The level of each cytokine was measured using technical duplicates.

### 2.6. Nitric Oxide (NO) Determination

RAW cells were seeded at 1 × 10^5^ cells per well in 96-well plates in complete media and treated for 24 h as indicated: SDF (500 µg/mL), LPS (1 µg/mL) and Tak-242 (10 µM). After treatment, 50 µL of extracellular media was collected from each well and transferred to a new 96-well plate to carry out the Griess reaction, according to the manufacturer’s instructions. NO is a short-lived free radical that is very difficult to detect in cells; one method to estimate NO levels is to determine the levels of nitrites, which are the oxidation products of NO [35]. A standard curve generated with known concentrations of nitrites was used to estimate the concentrations of nitrites in the extracellular media. The experiment was performed using technical duplicates.

### 2.7. Quantitative Reverse Transcriptase Real-Time Polymerase Chain Reaction (qRT-PCR)

The treated cells were detached using a cell scraper and washed with cold PBS. Total RNAs were isolated using the PureLink^®^ RNA Mini Kit (Invitrogen) according to the manufacturer’s instructions. The RNA was reverse-transcribed into cDNAs using the M0277 Reverse Transcription kit (New England BioLabs Inc.). The qRT-PCR reactions were set up using 5 ng cDNA per reaction, using the SYBR^®^ Green QRT-PCR Master Mix with Low ROX (Agilent Technologies, USA) and the primers described in Table 1

The PCR reaction was performed as follows: 95 °C for 10 min; 40 cycles consisting of 95 °C for 30 s, 60 °C for 1 min, and 72 °C for 1 min; a melting step consisting of 95 °C for 1 min, 55 °C for 30 s, and 95 °C for 30 s was added at the end of the cycles. ΔCt values were calculated using β-actin as the housekeeping gene. The fold of change was calculated as 2^Δ(ΔCt)^. The experiment was performed using technical duplicates.

### 2.8. Metabolic Activity Assay

RAW cells were seeded at 10,000 cells per well in 96-well plates in complete media and incubated for 24 h to allow for attachment. Six technical replicates were used for each condition. The experiment was repeated twice. Cells were further incubated for 24 h after the addition of either 500 μg/mL (final concentration) SDF or complete media. Changes in metabolic activity were then assessed by adding 1/10 volume 0.1 mg/mL of the metabolic dye Alamar Blue (AB) in each well [36]. After 6 h incubation, the fluorescence emission intensity of AB was measured at 590 nm (Ex: 540 nm). The fluorescence values from SDF-treated cells were compared to the fluorescence values of cells incubated with media only. The baseline fluorescence of the media was only subtracted from each fluorescence value before comparison.

### 2.9. Statistical Analysis

Six independent SDF preparations (biological replicates) were generated from cooked Pinto (Monterrey) bean flour, cooked Black (Eclipse) bean flour, raw Pinto (Monterrey) bean flour, and raw Black (Eclipse) bean flour. Each experiment was performed using these independent preparations. ELISA and NO assays were conducted in triplicate wells, and qPCR analyses were performed in technical duplicates. For the qPCR experiment, the SDF samples were named C1 to C6 and R1 to R6, or 101 to 124 (see Table A1 for details). In the graphs, mean values were plotted with standard deviations. ANOVA, followed by a Scheffe post hoc test, was used to calculate statistical differences between groups. These differences were indicated in the graphs with the presence of an asterisk (*) as follows: * *p* < 0.05; ** *p* < 0.01; *** *p* < 0.001. When indicated, Pearson’s correlation coefficients (R and *p*-value) were calculated using R (version 4.4.2; R Core Team, 2024). Correlation strengths were described as very strong (R > 0.8), strong (0.6 < R < 0.79) or moderate (0.4 < R < 0.59).

## 3. Results

### 3.1. Endotoxin Contents in SDF Samples

Endotoxin is a frequent contaminant in biological preparations and possesses macrophage-stimulating properties [33]. Therefore, each SDF sample was evaluated for its level of endotoxin using the chromogenic LAL assay, which is the gold standard for measuring endotoxins in biological samples [37]. The level of endotoxin in the SDF samples ranged from 0.08 to 0.12 ng/mL (Appendix A Table A1). It was previously reported that at least 0.5 ng/mL endotoxin was needed to induce in vitro cytokine production in macrophages and dendritic cells [38,39]. The levels of endotoxins in our SDF samples were below this concentration and were therefore not considered to significantly contribute to the observed increases in cytokines or NO levels in our biological assays.

### 3.2. SDF Stimulates the Production of NO by Macrophages in Part Through the Activation of the TLR-4 Signaling Pathway

We initially compared the levels of NO, TNF-α, IL-6 and IL-1β from RAW cells stimulated with Pinto/Monterrey SDF or Black/Eclipse SDF, but we did not observe statistically significant differences in NO or cytokine levels between the two varieties of bean. However, we observed differences in NO or cytokine levels between the SDF extracted from cooked or raw bean flours. For these reasons, for the rest of the study, the “cooked SDF” samples include those from Pinto/Monterrey and Black/Eclipse SDF. Similarly, the “raw SDF” samples include those from Pinto/Monterrey and Black/Eclipse SDF.

**Figure 1:**
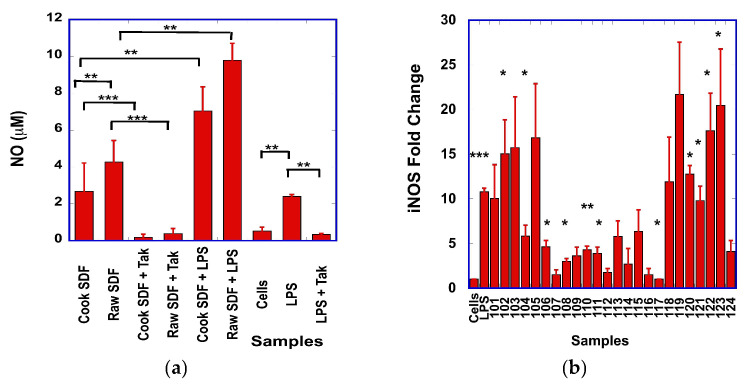
(**a**) Comparison of the NO levels produced from macrophages stimulated with raw or cooked SDF, with or without pre-stimulation with LPS, and in the presence or absence of the TLR-4 inhibitor Tak-242. Statistically significant differences in NO levels between different treatment groups are indicated by *: * *p* < 0.05; ** *p* < 0.01; *** *p* < 0.001. (**b**) Relative fold of changes in iNOS transcripts from unstimulated cells (Cells), cells stimulated with LPS (LPS), or cells stimulated with different raw (samples 101 to 112) or cooked (samples 113 to 124) SDF. For details on SDF nomenclature, see Appendix A Table A1. Statistically significant differences in mRNA levels between unstimulated cells and SDF-stimulated cells are indicated with *: * *p* < 0.05; ** *p* < 0.01; *** *p* < 0.001.

In our first set of experiments, we investigated whether the SDF could stimulate the production of NO by macrophages. Macrophage cell stimulation with LPS resulted in a statistically significant (*p* = 0.006) increase in NO levels (2.0 μM ± 0.03 μM) when compared to unstimulated cells (0.5 μM +/− 0.19 μM) (Figure 1a). Both raw and cooked SDF stimulated the production of NO when compared to unstimulated cells; NO levels were statistically lower (2.68 μM +/− 1.53 μM) from cooked SDF than from raw SDF (4.27 μM +/− 1.16 μM) (*p* = 0.009) (Figure 1a). Details on the effects of the different SDF on the production of NO by macrophages are presented in Appendix A (Figure A2).

Macrophages possess surface pattern recognition receptors (PRRs) that detect and bind to specific ligands known as pathogen-associated molecular patterns (PAMPs) or damage-associated molecular patterns (DAMPs); the activation of PRRs by PAMPs or DAMPs results in the upregulation and expression of inflammatory enzymes and cytokines [40]. Among PRRs, TLR-4 was found to mediate the activation of macrophages by polysaccharides [41]. When the macrophages were pre-incubated with the Tak-242 small molecule TLR-4 inhibitor [42] prior to stimulation with the SDF samples, we observed that NO production was statistically significantly reduced for both raw and cooked SDF samples (Figure 1a). These results suggest that the SDF triggers the production of NO from macrophages in part through the activation of the TLR-4 signaling pathway.

**Figure A1:**
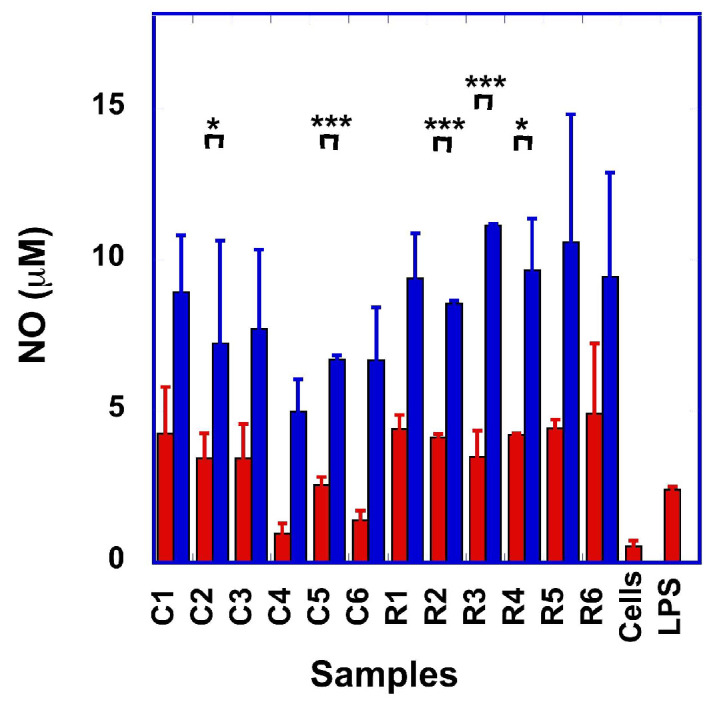
Comparison of the NO levels produced from macrophages stimulated with raw (R1–R6) or cooked (C1–C6) SDF, before (red bars) or after (blue bars) pre-stimulation with LPS. The statistical comparisons between the levels of NO generated from SDF-stimulated cells in the presence and absence of pre-stimulation with LPS are indicated. For SDF nomenclature, see Appendix A, Table A1 * *p* < 0.05; *** *p* < 0.001.

Because LPS stimulates the production of NO through the activation of TLR-4, we next investigated whether pre-incubation of the macrophages with LPS would affect the production of NO by the SDF. To our surprise, we observed that the NO levels were further increased when the macrophages were stimulated with SDF in the presence of LPS (Figure 1a and Appendix A, Figure A1). Overall, the levels of NO produced by LPS and SDF-stimulated macrophages were statistically significantly higher (*p* = 0.002) for raw SDF than for cooked SDF (Figure 1a). A strong positive Pearson correlation (R = 0.68; *p* = 0.002) was observed between the levels of NO produced by SDF in non-LPS-stimulated macrophages and in LPS-stimulated macrophages. In addition, we observed that, when considering all raw and all cocked SDF, the NO levels resulting from the stimulation with both SDF and LPS (7.04 μM +/− 1.31 μM for LPS and cooked SDF, and 9.79 μM +/− 0.092 μM for LPS and raw SDF) were higher than the sum of the NO levels obtained from the stimulation with SDF alone (2.68 μM +/− 1.53 μM for cooked SDF and 4.27 μM +/− 1.16 μM for raw SDF) and LPS alone (2.0 μM +/− 0.03 μM), suggesting a synergistic effect between SDF and LPS for the production of NO by macrophages (Figure 1a).

We next investigated if the NO levels generated from macrophages stimulated with SDF correlated with the levels of inducible (iNOS) mRNA. Stimulation of macrophages with LPS resulted in about a 10-fold increase in iNOS mRNA when compared with unstimulated cells (*p* < 0.001) (Figure 1b). Overall, the effects of the SDF on the levels of iNOS mRNA varied among SDF, with statistically significant (*p* < 0.05 to *p* < 0.01) differences observed with the cooked SDF samples 102, 104,106, 108, 110, 111, and raw SDF samples 120, 121, 122 and 123, when compared to unstimulated cells (Figure 1b). We performed a Pearson’s correlation analysis between the levels of NO produced by SDF-stimulated cells and the iNOS mRNA levels and observed a strong correlation (R = 0.71, *p* < 0.001), suggesting that SDF stimulates the production of NO through the upregulation of iNOS in macrophages.

### 3.3. SDF Stimulates the Production of TNF-α, IL-6 and IL-1β by Macrophages in Part Through the Activation of the TLR-4 Signaling Pathway

**Figure 2:**
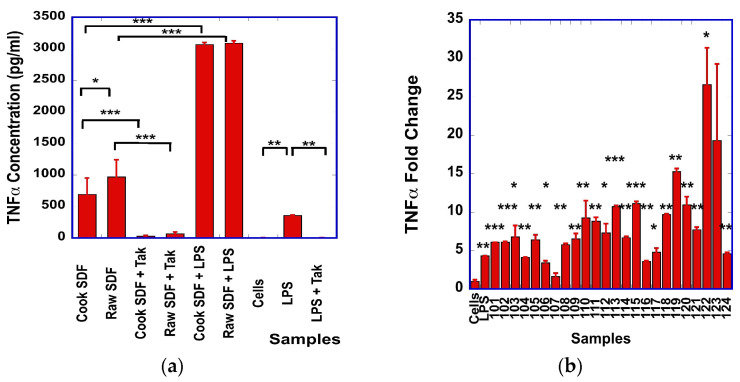
(**a**) Comparison of the TNF-α levels produced from cells stimulated with raw or cooked SDF, alone or in the presence of Tak-242, or after pre-stimulation with LPS. Statistically significant differences between treatment groups are indicated by *. (**b**) Relative fold of changes in TNF-α transcripts from unstimulated cells (Cells) or cells stimulated with LPS (LPS) or the different cooked (samples 101 to 112) or raw (samples 113 to 124) SDF. For SDF nomenclature, see Appendix A Table A1. Statistically significant differences in transcript levels between unstimulated and SDF-stimulated cells are indicated with *: * *p* < 0.05; ** *p* < 0.01; *** *p* < 0.001.

**Figure A2:**
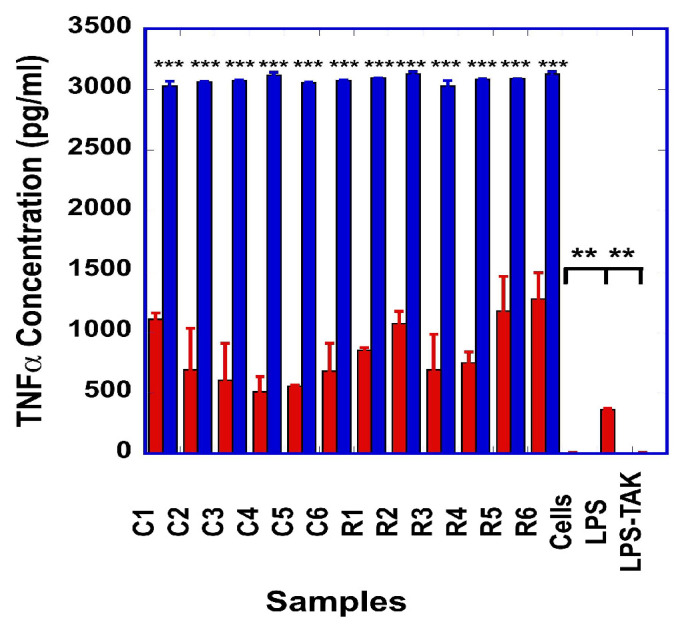
Comparison of TNF-α levels produced from macrophages stimulated with raw (R1–R6) or cooked (C1–C6) SDF, before (red bars) or after (blue bars) pre-stimulation with LPS. For SDF nomenclature, see Appendix A, Table A1. Statistical differences are indicated when observed. ** *p* < 0.01; *** *p* < 0.001.

We observed statistically higher levels of TNF-α in the supernatants of SDF-stimulated cells than in unstimulated cells (Figure 2a), with more than 100-fold difference in TNF-α levels between samples (Appendix A Figure A2). For seven of the SDF samples (C1, C2, R1, R2, R4, R5 and R6), we noted that the levels of TNF-α were statistically significantly higher than those generated by the cells stimulated by LPS alone (362 pg/mL +/− 10 pg/mL) (Appendix A Figure A2). The levels of TNF-α generated from cooked SDF samples were statistically significantly higher than from raw SDF (Figure 2a). Pre-incubation of the cells with Tak-242 resulted in a reduction in TNF-α production by the cells (Figure 2a), suggesting that the TLR-4 signaling pathway is involved in part in the generation of TNF-α from SDF-stimulated cells. When pre-incubating the RAW cells with LPS prior to the stimulation with SDF, we observed a 3-to-6-fold statistically significant higher level of TNF-α (Figure 2a) when compared to the cells treated with SDF alone, suggesting a synergistic effect between LPS and SDF in the macrophages.

The quantitative PCR analysis revealed large differences in TNF-α transcript levels, ranging from 1-fold to 25-fold, between the different SDF samples. Statistically significant differences between SDF-stimulated cells and unstimulated cells were observed in all but two SDF samples (Figure 2b). In addition, Pearson’s correlation analysis between the TNF-α levels, obtained by ELISA, and the mRNA fold changes showed a statistically significant moderate correlation (R = 0.58, *p* = 0.003).

**Figure 3:**
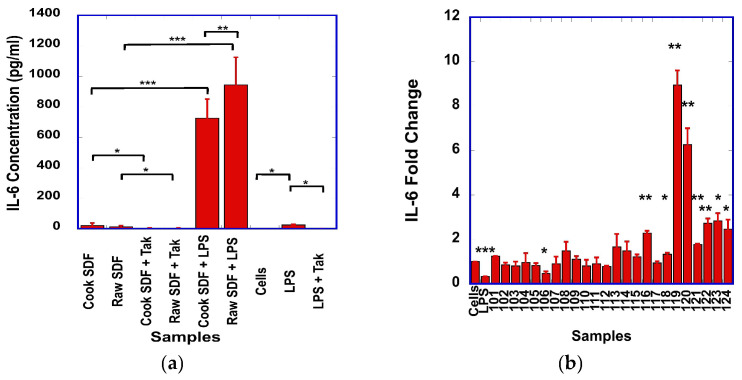
(**a**) Comparison of the IL-6 levels produced from cells stimulated with raw or cooked SDF, alone or in the presence of Tak-242, or after pre-stimulation with LPS. Statistically significant differences between treatment groups are indicated by *. (**b**) Relative fold of changes in IL-6 transcripts from unstimulated cells (Cells) or cells stimulated with LPS (LPS) or the different cooked (samples 101 to 112) or raw (samples 113 to 124) SDF. For SDF nomenclature, see Appendix A Table A1. Statistically significant differences in transcript levels between unstimulated and SDF-stimulated cells are indicated with *: * *p* < 0.05; ** *p* < 0.01; *** *p* < 0.001.

Stimulation of the macrophages with the bean SDF also resulted in increased IL-6 (Figure 3a). No difference in IL-6 levels was observed between RAW cells stimulated with cooked and raw SDF. However, after pre-stimulation with LPS, the IL-6 levels generated from raw SDF were higher than those generated from cooked SDF (Figure 3a).

**Figure A3:**
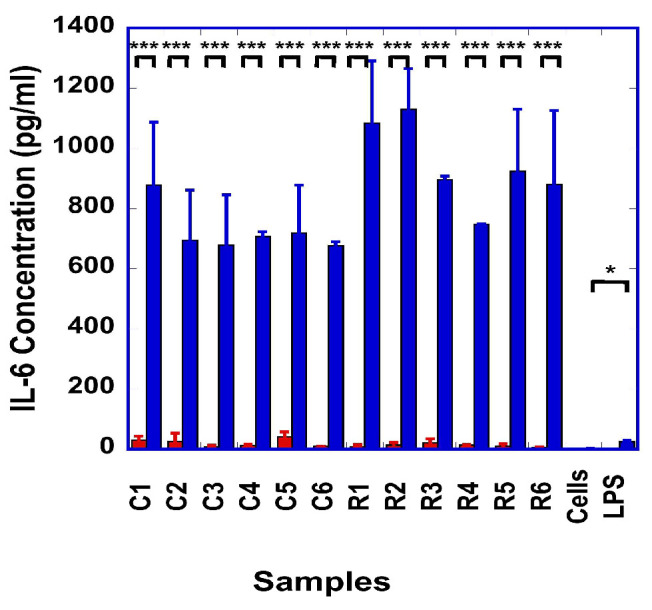
Comparison of IL-6 levels produced from macrophages stimulated with raw (R1–R6) or cooked (C1–C6) SDF, before (red bars) or after (blue bars) stimulation with LPS. For SDF nomenclature, see Appendix A, Table A1. Statistical differences are indicated when observed. * *p* < 0.05; *** *p* < 0.001.

For example, the macrophages produced from 17-fold (observed with C5) up to 145-fold (observed with R6) statistically significantly higher levels of IL-6, in the presence of LPS and SDF, than when stimulated with SDF alone, suggesting a very strong synergistic effect between the SDF and LPS (Appendix A Figure A3). After pre-treatment with Tak-242, the levels of IL-6 produced from SDF-treated cells significantly decreased, suggesting a role of the TLR-4 signaling pathway in the production of IL-6 (Figure 3a).

IL-6 transcript levels were found to increase with the stimulation from one cooked (106) and eight different raw samples (116, 118, 119, 120, 121, 122, 123 and 124) (Figure 3b). However, surprisingly, no statistically significant correlation (R = 0.17, *p* = 0.420) was observed between the transcript and protein levels of IL-6.

**Figure 4:**
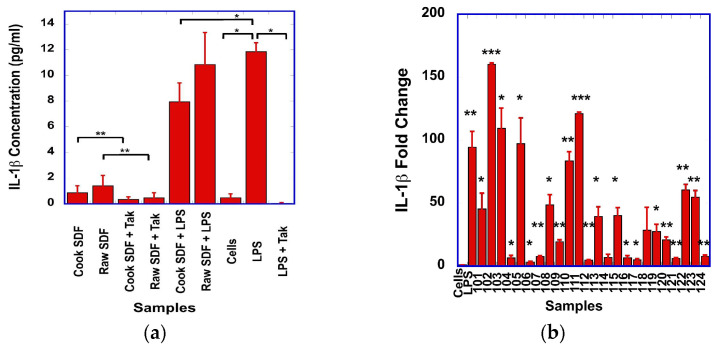
(**a**) Comparison of the IL-1β levels produced from cells stimulated with raw or cooked SDF, alone or in the presence of Tak-242, or after pre-stimulation with LPS. Statistically significant differences between treatment groups are indicated by *. (**b**) Relative fold of changes in IL-1β transcripts from unstimulated cells (Cells) or cells stimulated with LPS (LPS) or the different cooked (samples 101 to 112) or raw (samples 113 to 124) SDF. For SDF nomenclature, see Appendix A Table A1. Statistically significant differences in transcript levels between unstimulated and SDF-stimulated cells are indicated with *: * *p* < 0.05; ** *p* < 0.01; *** *p* < 0.001.

The effect of SDF on the production of IL-1β by macrophages was variable among SDF, without statistically significant differences when compared to unstimulated cells. In general, SDF stimulation resulted from lower levels of IL-1β than the stimulation with LPS (Figure 4a). However, the levels of IL-1β from SDF-stimulated cells were reduced when cells were pre-treated with Tak-242, for both cooked and raw SDF (Figure 4a).

**Figure A4:**
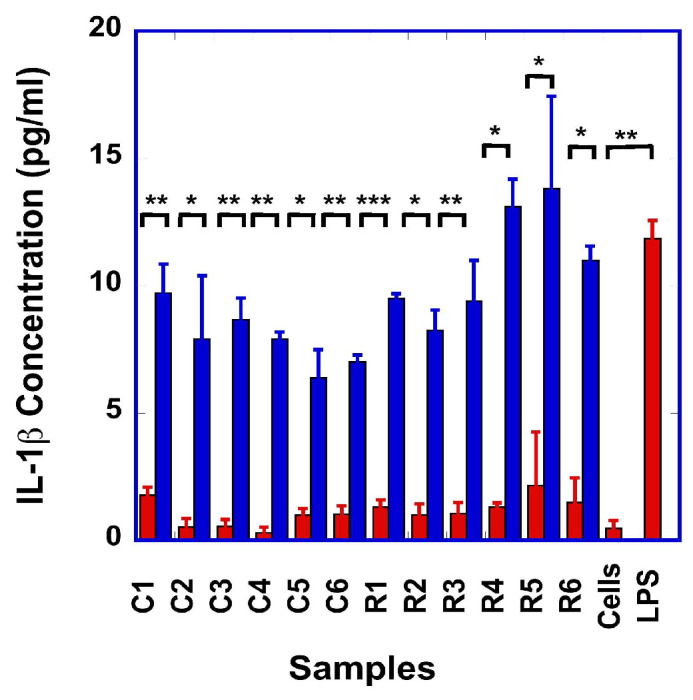
Comparison of IL-1β levels produced from macrophages stimulated with raw (R1–R6) or cooked (C1–C6) SDF, before (red bars) or after (blue bars) stimulation with LPS. For SDF nomenclature, see Appendix A, Table A1. Statistical differences are indicated when observed. * *p* < 0.05; ** *p* < 0.01; *** *p* < 0.001.

Pre-incubation of the cells with LPS resulted in large increases in IL-1β for both cooked and raw SDF (Figure 4a). However, these levels were lower than those generated from cells stimulated with LPS alone (Figure 4a and Appendix A Figure A4).

At the transcript level, all but two SDF samples showed statistically significant increases in IL-1β when compared to unstimulated cells (Figure 4b). Levels of IL-1β transcripts from SDF-stimulated cells were higher than from unstimulated cells but generally lower than from LPS-stimulated cells. For half the SDF samples, the levels of IL-1β transcripts were increased by more than 40-fold compared to those from unstimulated cells (Figure 4b). Interestingly, these large differences in IL-1β transcript levels were not observed at the protein level, where only about 2-fold changes in IL-1β were observed among SDF. In line with this observation, no Pearson correlation was observed between the transcript and the protein levels of IL-1β.

**Figure 5:**
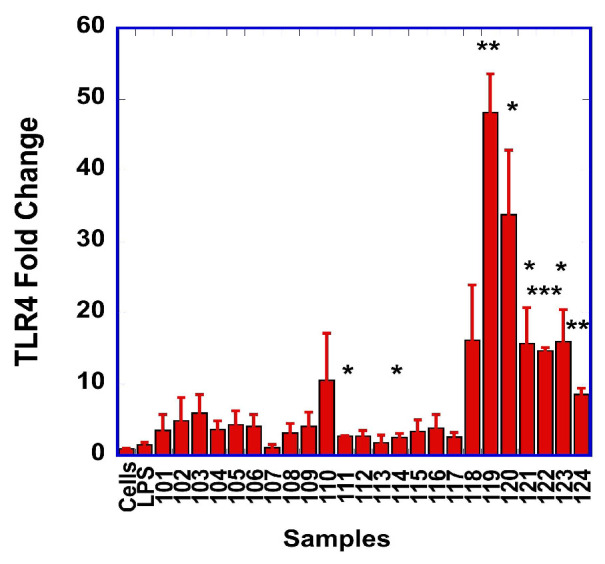
Relative fold of changes in TLR-4 transcripts from unstimulated cells (Cells) or cells stimulated with LPS (LPS), or the different cooked (samples 101 to 112) or raw (samples 113 to 124) SDF. For SDF nomenclature, see Appendix A Table A1. Statistically significant differences in transcript levels between unstimulated and SDF-stimulated cells are indicated with *: * *p* < 0.05; ** *p* < 0.01; *** *p* < 0.001.

After observing that the TLR-4 signaling pathway was involved in the production of NO and of the pro-inflammatory cytokines TNF-α, IL-6 and IL-1β, we investigated if the stimulation of the macrophages with the SDF resulted in changes in TLR-4 transcript levels (Figure 5). We observed statistically significant increases in TLR-4 transcripts with one cooked (111) sample and seven raw (114, 119, 120, 121, 122, 123 and 124) samples.

Pearson’s correlation analysis between TNF-α and TLR-4 transcripts revealed a moderate correlation (R = 0.51, *p* = 0.011), suggesting a link between TNF-α generated from the cells and the upregulation of TLR-4.

## 4. Discussion

Many studies have reported that plant-derived polysaccharides promote the production of pro-inflammatory cytokines by macrophages. Examples of plants include the herbaceous vine *Tinosporia cordifolia*, elderberries (*Sambucus nigra* L.), an orchidaceae plant (*Dendrobium huoshanense*) and the black nightshade plant (*Solanum nigrum*) [32,43,44]. In these studies, the plant-derived polysaccharides stimulated the macrophages through their interaction with TLR-4 [32,43,44].

We observed that dry bean-derived SDF has similar immunomodulatory effects to other plant-derived polysaccharides. In our experimental conditions, we showed that the SDF prepared from dry beans stimulated the production of NO (Figure 1a), as well as the expression of TNF-α (Figure 2a), IL-1β (Figure 4a) and IL-6 (Figure 3a) by the RAW macrophages. A strong correlation was found between the transcript levels of iNOS and NO levels, suggesting that iNOS was involved in the production of NO.

When evaluating Pearson correlations between the protein and transcript levels of the three examined cytokines, we only observed a strong Pearson correlation between the protein and transcript levels for TNF-α; no statistically significant correlations were found between the protein and transcript levels of IL-1β and IL-6. This discrepancy could be the result of complex post-transcriptional mechanisms regulating the stability of IL-1β and IL-6 transcripts. Indeed, both IL-6 and IL-1β transcripts are unstable, and their transcription is tightly regulated to prevent excessive inflammation [45,46]. Additional mechanisms could involve differences in inflammasome activity or protein degradation [47]. For instance, it was shown that in dendritic cells, IL-6 signaling results in auto-inhibition of IL-6 synthesis, in order to prevent excessive production of IL-6 by cells [48]. Similarly, complex mechanisms regulate the secretion of IL-1β. When synthesized in cells, IL-1β is produced in the form of a precursor protein or pro-IL-1β, which is converted into active IL-1β through a proteolytic cleavage performed by intracellular NLR family pyrin domain-containing 3 (NLRP3)/Apoptosis-associated Speck-like protein containing a CARD (ASC)/caspase-1 inflammasomes in the presence of activating signals [49,50]. In the absence of these activating signals, pro-IL-1β is not converted into the active IL-1β, and might either remain unprocessed intracellularly or be degraded following ubiquitination [51,52]. These complex mechanisms might explain the absence of a positive correlation between the levels of transcript and of secreted protein for these two cytokines (IL-1β and IL-6). Additional experiments would be necessary to determine these mechanisms.

We showed in our study that the synthesis of NO and the production of the pro-inflammatory cytokines (TNF-α, IL-1β and IL-6) by the RAW macrophages were strongly reduced by Tak-242, a small molecule inhibitor of TLR-4. This suggests that the pro-inflammatory effects of the SDF are mediated in part by the TLR-4 signaling pathway. Interestingly, we also observed that 8 out of the 24 SDF stimulated the transcription of TLR-4; one cooked SDF (#111) and seven raw SDF (#114, #119, #120, #121, #122, #123, and #124). The strong Pearson correlation between the transcript levels of TLR-4 and TNF-α further supports the role of TLR-4 in mediating the pro-inflammatory effects of the SDF. Studies have shown that alginate-derived guluronate oligosaccharides also stimulated the transcription and expression of TLR-4, which is in line with our observations [53]. However, we acknowledge that other pattern recognition receptors, such as TLR-2 and Dectin-1, could be involved in the pro-inflammatory response from the RAW macrophages. Indeed, polysaccharides extracted from *Acanthopanax senticosus* were shown to activate TLR-2 [54].

As it was previously reported that plant-derived polysaccharides could bind to and compete with LPS for binding to TLR-4 [54,55], we anticipated that the addition of SDF to LPS-stimulated macrophages would result in a decreased production of NO and pro-inflammatory cytokines. However, we observed a synergistic pro-inflammatory effect between LPS and SDF. Interestingly, Ferwerda et al. recently reported that bacterial β-glucans and LPS could synergistically activate TLR-4. In addition, these authors showed that when bound to β-glucans, Dectin 1 could also synergize with both TLR-4 and TLR-2 [56]. Further studies using neutralizing antibodies against TLR-4, TLR-2, as well as Dectin-1 could be performed to elucidate the pro-inflammatory signaling pathways activated by the SDF. Additional studies using RAW cells lacking these pattern recognition receptors could further inform on these signaling pathways.

**Figure A5:**
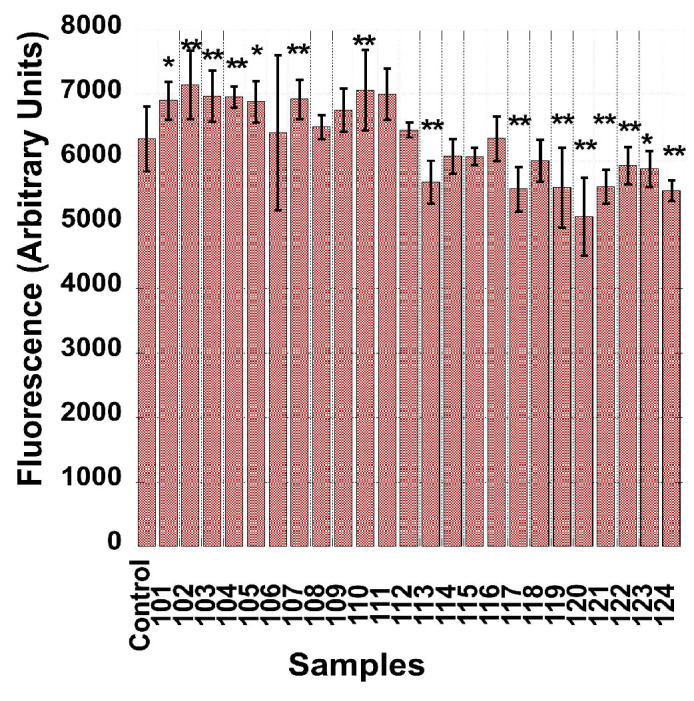
Changes in metabolic activity of RAW cells after 24h incubation with 500 μg/mL SDF. Control represents cells incubated with complete media only. For SDF nomenclature, see Appendix A, Table A1. * Indicates the statistical difference between control cells and cells incubated with SDF. * *p* < 0.05; ** *p* < 0.01.

One of the goals of our study was to determine if the process of cooking the dry bean flour had some effect on the immunomodulatory properties of the SDF. We observed statistically significantly higher levels of NO and TNF-α in RAW macrophages stimulated with raw SDF than with cooked SDF (Figure 1a and Figure 2a). These effects were not observed for the expression of IL-1β and IL-6. We also observed higher macrophage metabolic activity with cooked SDF than with raw SDF (Figure A5). All but three types of SDF derived from cooked flour show a moderate (up to 12%) increase in metabolic activity at 500 µg/mL compared to control cells. Similarly, all but three types of SDF derived from raw flour showed moderately lower (up to 19% lower) metabolic activity than untreated cells. Our data suggest that cooking dry bean flour could alter the immunomodulatory properties of SDF extracted from the flour.

The chemical and physical properties of the SDF extracted from the raw and cooked dry bean were characterized in a previously published manuscript [33]. The extracts contained pectin-rich polysaccharides (mainly arabinose- and galacturonic acid-based structures), hemicelluloses (xyloglucans, xylans), and raffinose family oligosaccharides (RFOs). Molecular weight analysis of the dry bean-derived SDF revealed two fractions, a high-molecular-weight (HMW) component (≈4800–5000 kDa) dominated by stable pectin polymers and a low-molecular-weight (LMW) component (<3000 kDa) comprising shorter pectins and RFOs. Cooking reduced the LMW fraction, consistent with RFO depolymerization.

The observed differences between raw and cooked SDF-rich fractions suggest that thermal processing alters structural features that influence macrophage activation. Cooking is known to disrupt plant cell wall architecture, increasing the solubility and extractability of polysaccharides such as pectins and hemicelluloses. This enhanced extractability may improve the accessibility of bioactive components to immune cells. In addition, thermal processing can modify the molecular-weight distribution and conformation of polysaccharides, potentially generating fractions with greater biological activity. Changes in branching patterns or the exposure of functional groups, such as uronic acids and neutral sugar side chains, may enhance interactions with pattern recognition receptors, including TLR-4. Furthermore, cooking may alter the association between dietary fiber and phenolic compounds, either by releasing bound phenolics or modifying fiber–phenolic complexes, which could contribute to the observed immunomodulatory effects. Together, these structural and compositional changes provide a plausible explanation for the greater macrophage activation induced by cooked SDF-rich fractions compared to raw counterparts. Future experiments would include examining in detail the effect of heat-processing on the structural characteristics of dry bean-derived SDF and their possible correlation with immune modulatory functions.

When comparing the immunomodulatory functions of dry bean extracts, it is important to consider the protein levels of the biological samples. For example, when prepared as protein hydrolyzates and not SDF, dry bean extracts from *Phaseolus vulgaris* L. were shown to inhibit LPS-induced NO production in RAW 264.7 macrophages in a dose-dependent manner [30,57]. Similar observations were reported by López-Barrios et al. [57]. In the study of López-Barrios et al., the levels of NO produced from LPS-stimulated macrophages were found to be further reduced following enzymatic digestion of the protein hydrolyzates, suggesting that smaller peptides were more biologically active than larger peptides [57].

Protein hydrolyzates derived from dry beans typically contain 70% protein or peptide [58]. In our study, the SDF samples contained only between 13.8% and 21.3% residual protein [27]. Cooking significantly increased sugar content while reducing residual protein, likely due to enhanced proteolysis. The level of protein or peptide content in SDF samples could thus modulate the balance between anti- and pro-inflammatory effects in macrophages. It is also noteworthy that the cited studies above [30,57] did not utilize whole-grain flour to prepare the protein hydrolyzates, as the beans were dehulled. Bean hulls are the part of the seed with the highest fiber content, so it can be assumed that their protein hydrolyzate fractions did not contain fibers that may elicit the results we have described.

The presence of phenolic compounds in the dry bean extracts could also influence the experimental outcomes. In one study, dry bean phenolic-rich extracts were shown to have anti-inflammatory properties by reducing the levels of NO and iNOS, TNF-α, IL-1β and IL-6 transcripts, in LPS-stimulated RAW macrophages [29]. In another study, phenolic extracts obtained from the seed coats (hulls) of dry beans were also shown to have anti-inflammatory properties by inhibiting cyclooxygenases (COX) 1 and 2, as well as lipoxygenase (LOX) [59]. Similar anti-inflammatory effects with inhibition of COX and LOX enzymes have been reported with phenolic extracts from faba beans (*Vicia faba* L.) [60]. These data further suggest that the presence of phenolic compounds in SDF samples might also affect the inflammatory response in macrophages. The SDF samples used in our study may contain certain levels of phenols, such as flavonoids, kaempferol 3-0 glucoside, hydroxycinnamic acid derivatives or anthocyanins, since the whole grain flours from which the SDF samples were extracted contained these compounds [27]. These co-extracted components could independently influence macrophage activation and represent a key limitation of our study, as they could have interfered with the outcomes by counteracting the pro-inflammatory effects. Therefore, in future studies, quantification and characterization of the phenolic compounds in the SDF samples should be conducted to fully determine their impact.

## 5. Conclusions

In summary, this study demonstrates that SDF-rich fractions derived from dry beans modulate macrophage activity in vitro, promoting a pro-inflammatory response characterized by increased production of nitric oxide and cytokines. The observed effects were partially attenuated by TLR-4 inhibition, suggesting involvement of this signaling pathway, although additional mechanisms may also contribute.

Importantly, the SDF-rich fractions used in this study are heterogeneous and contain residual non-fiber components, such as proteins and phenolic compounds, which may have influenced the observed responses. Furthermore, as this work was conducted in RAW 264.7 macrophages, the findings are limited to an in vitro model and may not directly translate to physiological conditions.

Future studies should focus on the use of more refined fractions, evaluating their mechanisms of action, and determining their physiological relevance in more complex biological systems to better understand the immunomodulatory potential of bean-derived components.

## Figures and Tables

**Table 1:** Primer sequences used in the study.

Gene	Primer Direction	Sequence 5′-3′
β actin	Forward	CATGTACGTTGCTATCCAGGC
β actin	Reverse	CTCCTTAATGTCACGCACGAT
TNF-α	Forward	GACGTGGAACTGGCAGAAGAG
TNF-α	Reverse	TTGGTGGTTTGTGAGTGTGAG
IL-1β	Forward	GAAATGCCACCTTTTGACAGTG
IL-1β	Reverse	TGGATGCTCTCATCAGGACAG
IL-6	Forward	CCAAGAGGTGAGTGCTTCCC
IL-6	Reverse	CTGTTGTTCAGACTCTCTCCCT
iNOS	Forward	GTTCTCAGCCCAACAATACAAGA
iNOS	Reverse	GTGGACGGGTCGATGTCAC
TLR-4	Forward	ATATTGACAGGAAACCCCATCCA
TLR-4	Reverse	TAGAACCCGCAAGTCTGTGC

## Data Availability

The original contributions presented in this study are included in the article. Further inquiries can be directed to the corresponding author.

## Abbreviations

SDF	Soluble dietary fiber
NO	Nitric oxide
TNF	Tumor necrosis factor
IL	Interleukin
TLR	Toll-like receptor
DF	Dietary fiber
IDF	Insoluble dietary fiber
NK	Natural killer
INF	Interferon
LPS	Lipopolysaccharide
AXH	Arabinoxylan hydrolyzate
FBS	Fetal bovine serum
LAL	Limulus amebocyte lysate
DMEM	Dulbecco’s modified Eagle’s medium
ND	North Dakota
qRT-PCR	Quantitative reverse transcriptase real-time polymerase chain reaction
AB	Alamar Blue
iNOS	Inducible nitric oxide synthase
PRR	Pattern recognition receptor
PAMP	Pathogen-associated molecular pattern
DAMP	Damage-associated molecular pattern
NLRP3	NLR family pyrin domain-containing 3
ASC	Apoptosis-associated Speck-like protein containing a CARD
RFOs	Raffinose family oligosaccharides
HMW	High molecular weight
LMW	Low molecular weight
COX	Cycloxygenase
LOX	Lipoxygenase

## Appendix A

**Table A1:** Details of the SDF samples used in the study. The endotoxin level (ng/mL) was measured in each of the samples. Six independent SDF samples were prepared from cooked Pinto/Monterrey flour and are identified as C1.1 to C3.2, or 101 to 106. Six independent SDF samples were prepared from cooked Black/Eclipse flour and are identified as C4.1 to C6.2, or 107 to 112. Six independent SDF samples were prepared from raw Pinto/Monterrey flour and are identified as R1.1 to R3.2, or 113 to 118. Six independent SDF samples were prepared from RAW Black/Eclipse flour and are identified as R4.1 to R6.2, or 118 to 124.

Sample Number		Type/Variety	Growth Location	Process	Endotoxin Level (ng/mL)
C1.1	101	Pinto/Monterrey	Hatton	Cooked	0.120
C1.2	102	Pinto/Monterrey	Hatton	Cooked	0.122
C2.1	103	Pinto/Monterrey	Prosper	Cooked	0.121
C2.2	104	Pinto/Monterrey	Prosper	Cooked	0.119
C3.1	105	Pinto/Monterrey	Forest River	Cooked	0.109
C3.2	106	Pinto/Monterrey	Forest River	Cooked	0.121
C4.1	107	Black/Eclipse	Hatton	Cooked	0.120
C4.2	108	Black/Eclipse	Hatton	Cooked	0.122
C5.1	109	Black/Eclipse	Prosper	Cooked	0.119
C5.2	110	Black/Eclipse	Prosper	Cooked	0.117
C6.1	111	Black/Eclipse	Forest River	Cooked	0.120
C6.2	112	Black/Eclipse	Forest River	Cooked	0.109
R1.1	113	Pinto/Monterrey	Hatton	Raw	0.120
R1.2	114	Pinto/Monterrey	Hatton	Raw	0.121
R2.1	115	Pinto/Monterrey	Prosper	Raw	0.105
R2.2	116	Pinto/Monterrey	Prosper	Raw	0.118
R3.1	117	Pinto/Monterrey	Forest River	Raw	0.101
R3.2	118	Pinto/Monterrey	Forest River	Raw	0.103
R 4.1	119	Black/Eclipse	Hatton	Raw	0.09
R4.2	120	Black/Eclipse	Hatton	Raw	0.08
R5.1	121	Black/Eclipse	Prosper	Raw	0.120
R5.2	122	Black/Eclipse	Prosper	Raw	0.120
R6.1	123	Black/Eclipse	Forest River	Raw	0.115
R6.2	124	Black/Eclipse	Forest River	Raw	0.102
